# Translation of circHGF RNA encodes an HGF protein variant promoting glioblastoma growth through stimulation of c-MET

**DOI:** 10.1007/s11060-023-04331-5

**Published:** 2023-05-10

**Authors:** Jacquelyn T. Saunders, Sunil Kumar, Angelica Benavides-Serrato, Brent Holmes, Kennedy E. Benavides, Muhammad T. Bashir, Robert N. Nishimura, Joseph Gera

**Affiliations:** 1 Department of Medicine, David Geffen School of Medicine at UCLA, University of California-Los Angeles, Greater Los Angeles Veterans Affairs Healthcare System, 16111 Plummer Street (151), Building 1, Room C111A, Los Angeles, CA 91343 USA; 2 Department of Neurology, David Geffen School of Medicine at UCLA, University of California-Los Angeles, Los Angeles, USA; 3 Jonnson Comprehensive Cancer Center, University of California-Los Angeles, Greater Los Angeles Veterans Affairs Healthcare System, Greater Los Angeles Veterans Affairs Healthcare System, 16111 Plummer Street (151), Building 1, Room C111A, Los Angeles, CA 91343 USA; 4 Molecular Biology Institute, University of California-Los Angeles, Greater Los Angeles Veterans Affairs Healthcare System, 16111 Plummer Street (151), Building 1, Room C111A, Los Angeles, CA 91343 USA; 5 Department of Research & Development, Greater Los Angeles Veterans Affairs Healthcare System, 16111 Plummer Street (151), Building 1, Room C111A, Los Angeles, CA 91343 USA

**Keywords:** Glioblastoma, Circular RNA, HGF, Translation, c-MET

## Abstract

**Introduction:**

HGF/c-MET signaling is a significant driver of glioblastoma (GBM) growth and disease progression. Unfortunately, c-MET targeted therapies have been found to be largely ineffective suggesting additional redundant mechanisms of c-MET activation.

**Methods:**

Utilizing RNA-sequencing (RNA-seq) and ribosome profiling analyses of circular RNAs, *circ-HGF* (*hsa_circ_0080914)* was identified as markedly upregulated in primary GBM and found to potentially encode an HGF protein variant (C-HGF) 119 amino acids in length. This candidate HGF variant was characterized and evaluated for its ability to mediate c-MET activation and regulate PDX GBM cell growth, motility and invasive potential in vitro and tumor burden in intracranial xenografts in mice.

**Results:**

An internal ribosome entry site (IRES) was identified within the *circ-HGF* RNA which mediated translation of the cross-junctional ORF encoding C-HGF and was observed to be highly expressed in GBM relative to normal brain tissue. C-HGF was also found to be secreted from GBM cells and concentrated cell culture supernatants or recombinant C-HGF activated known signaling cascades downstream of c-MET. C-HGF was shown to interact directly with the c-MET receptor resulting in its autophosphorylation and activation in PDX GBM lines. Knockdown of C-HGF resulted in suppression of c-MET signaling and marked inhibition of cell growth, motility and invasiveness, whereas overexpression of C-HGF displayed the opposite effects. Additionally, modulation of C-HGF expression regulated tumor growth in intracranial xenografted PDX GBM models.

**Conclusions:**

These results reveal an alternative mechanism of c-MET activation via a circular RNA encoded HGF protein variant which is relevant in GBM biology. Targeting C-HGF may offer a promising approach for GBM clinical management.

**Supplementary Information:**

The online version contains supplementary material available at 10.1007/s11060-023-04331-5.

## Introduction

Glioblastoma is a highly lethal CNS cancer with a median survival of only 12–17 month [1, 2]. Unfortunately, notwithstanding a relatively progressive understanding of the mutational landscape of the disease, genomic insights have failed to yield improvements in overall patient survival [3, 4]. Several factors contribute to therapeutic failure, notably poor blood–brain barrier penetration of current targeted inhibitors and intratumoral heterogeneity with high plasticity limit effectiveness of current therapies [5, 6]. Recently, amplified extrachromosomal DNA containing oncogenes have also been demonstrated to play a role in targeted therapy resistance [7]. c-MET amplification occurs in 47% of primary and 44% of secondary GBM [8]. Moreover, activating mutations in c-MET are significant events during the progression of low-grade gliomas to secondary GBM [9]. An analysis of TCGA data demonstrated that ~ 30% of GBM overexpress the ligand for c-MET, hepatocyte growth factor (HGF), and c-MET, suggesting autocrine activation may occur in patients [10]. Efforts to target c-MET as monotherapy or in combination with other therapies have been unsuccessful in GBM therapy [11]. While poor BBB penetration and intratumoral heterogeneity may provide some explanation as to the lack of effectiveness of c-MET targeted therapies, other unidentified modulators may also participate in GBM growth and therapy resistance.

Circular RNAs (circRNAs) are covalently closed RNA transcripts which are generally expressed at reduced levels relative to their linear cognate mRNAs. Several circRNAs have been shown to be translated and the protein products demonstrated to have important roles in cancer [12]. In fact, most circRNA translation products to date have effects on tumor cell progression or harbor tumor suppressor activities, consequently these peptides/short proteins represent promising drug targets for tumor treatment as well as potential biomarkers [13]. Perhaps most striking is the observation that the majority of the circRNA translation products described play important roles in glioma tumorigenesis and malignant progression indicating the relevance of this novel class of RNAs in this neoplasm [13, 14]. CircRNAs have also been found to be exceptionally stable as compared to their linear counterparts [12, 15].

Here we report the discovery of a circRNA-templated HGF protein variant derived from IRES-mediated translation of the *circ-HGF* RNA. This protein variant (C-HGF) is secreted from GBM cells and stimulates c-MET activity and its downstream signaling effectors. Modulation of C-HGF expression in PDX cell line models demonstrated regulation of cell growth, motility and invasive characteristics and markedly affected in vitro tumor growth in xenograft experiments.

## Materials and methods

Details regarding cell cultures, reagents, in vitro and  in vivo protocols and data analyses are described in Online Resource 1 Supplemental Materials and Methods.

## Results

### *Circular-HGF* is a potential coding RNA in glioblastoma

**Fig. 1:**
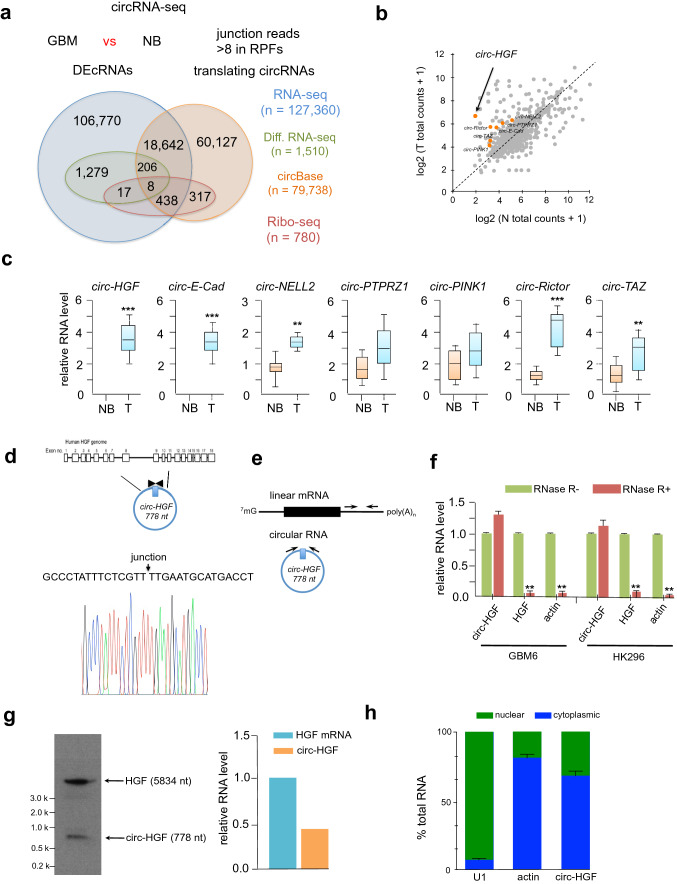
Circular-HGF is a candidate coding circRNA. **a** Approach undertaken for circRNA-sequencing (RNA-seq) and ribosome profiling (Ribo-seq) experiments. 4-paired GBM and normal brain (NB) were subjected to circ-RNA seq and Ribo-seq. Shown is a Venn diagram of coding circRNAs and differentially expressed circular RNAs (DEcRNAs) overlapping in GBM and NB. **b** Differentially expressed circular RNAs from Ribo-seq experiments. Candidate upregulated circRNAs are labeled orange. **c** Relative expression levels as determined via qRT-PCR of protein encoding circRNAs versus NB. *n* = 14 independent samples and results are shown as box plots containing the 1st and 3rd quartiles. Whiskers indicate minima and maxima. Wilcoxon test, **, *p* = 0.005, ***, *p* < 0.001. **d** Illustration of the annotated genomic region of the human HGF gene and derived *circ-HGF* RNA [36, 37]. Sanger sequencing was conducted to confirm head-to-tail splicing from GBM cells. **e** Schematic of convergent and divergent primer design to detect linear and circular HGF RNAs. **f** *Circ-HGF* RNA is resistant to RNase-R treatment from GBM6 or HK296 PDX GBM cells. **, *p* < 0.05, *n* = 3. **g** Northern blot analysis was performed to determine the RNA the relative levels of linear and circular RNAs for HGF in GBM6 cells. **h** The relative expression of *circ-HGF* was assessed by qRT-PCR in nuclear and cytoplasmic fractions from GBM6 cells. *n* = 3

To identify differentially expressed circRNAs in GBM we compared primary and normal tissue from 4-paired sets of samples by RNA-seq analyses (Fig. 1A). A total of 127,360 circRNAs were identified, of which 19,294 had been previously annotated in circBase [16]. A majority of circRNAs were less then 1000 nucleotides in length and of the 1510 confirmed differentially expressed circular RNAs, 1296 were significantly downregulated, while 214 were markedly upregulated (by a factor of 2.5 fold relative to normal brain). We also performed ribosome profiling on these 4-paired GBM versus normal brain samples to identify circRNAs which were translatable (Fig. 1B). We concentrated our efforts on identifying head-to-tail junction reads which were specific for translating circRNAs. We excluded any reads containing mismatches and confined the minimum read-junction overlap to eight nucleotides on either side of the junction site. Potential translating circRNAs were chosen when the unique junction reads were found in at least three of the samples and more then eight total junction reads were observed. We identified a total of 780 high confidence circRNAs, of which 763 were annotated in circBase. We then compared these coding circRNAs to the differentially expressed circRNAs and identified 25 circRNAs which were differentially translated between GBM and normal brain samples (Online Resource 2 Suppl. Fig. S1). Seven potential candidate circRNAs, which were upregulated in GBM, were validated by qRT-PCR (Fig. 1C). As GBM samples may include other cell types which may introduce expression biases, we also confirmed these findings in a panel of GBM PDX lines (GBM6, GBM9, GBM43, HK296; lines expressed c-MET) (Online Resource 3 Suppl. Fig. S2) and we identified *circ-HGF* (*hsa_circ_0080914*) as one of the most differentially upregulated circRNAs which we selected for further characterization as HGF/c-MET activation is a known driver of GBM [11, 17]. As annotated in circBase using the human reference genome GRCh37/hg19, *circ-HGF* is generated by the backsplicing of exons 6–11 of HGF located on chromosome 7 in GBM (Fig. 1D). Convergent and divergent primers were designed to detect linear mRNA and circular RNA (Fig. 1E) and subsequently utilized to monitor these species in RNase-R treated RNA samples from GBM6 or HK296 cells. As shown in Fig. 1F, following treatment with RNase-R, linear HGF mRNA displayed a significant reduction in abundance whereas, *circ-HGF* was relatively resistant consistent with its closed circular structure and increased stability as compared to its cognate mRNA. Northern blotting was also performed to determine the relative RNA levels of HGF mRNA and *circ-HGF* (Fig. 1G). Subsequent qRT-PCR analysis of nuclear and cytoplasmic cell fractions displayed elevated cytoplasmic localization of *circ-HGF* RNA (Fig. 1H) consistent with its potential translation.

### *Circ-HGF* encodes a 119 amino acid protein which is translated by an IRES and is overexpressed in GBM

**Fig. 2:**
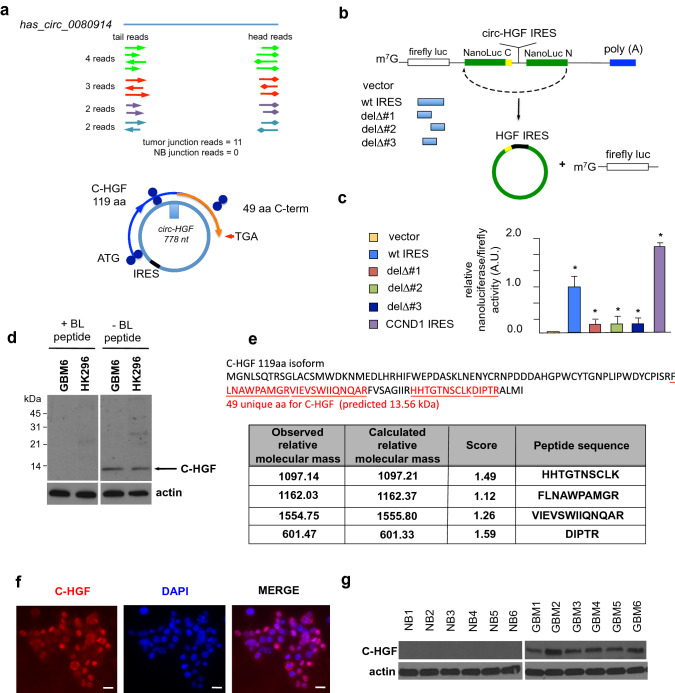
*Circ-HGF* is translated via an IRES encoding a 119 amino-acid product which is highly expressed in GBM. **a** *Top,* Ribosome footprints in the *circ-HGF* RNA junction. Four GBM had 11 junction reads while none were detected in four NB samples. *Bottom*, illustration of IRES-mediated C-HGF translation product from *circ-HGF* containing a cross-junction unique 49 residue C-terminus. **b** Strategy employed for verification of the *circ-HGF* IRES utilizing a dual luciferase-split nanoluciferase construct. **c** GBM6 cells were transiently transfected with the *wt* IRES, the indicated IRES deletion mutants or a positive control CCND1 IRES construct and Firefly and NanoLuc activities determined. Mean + S.D., *n* = 3., *, *p* < 0.05. **d** Detection of a endogenous 13.6 kDa C-HGF protein product by antibodies specific for the 49 aa C-terminus. GBM6 or HK296 cell extracts were immunoblotted for C-HGF in the presence or absence of a specific blocking peptide. **e** Mass spectrometry analyses of protein samples from GBM6 following immunoprecipitation utilizing C-HGF specific antibodies. Shown are specific peptides identified from the unique C-terminal protein sequence of C-HGF. **f** Indirect immunofluorescence images of C-HGF expression in primary GBM utilizing C-HGF specific antibodies and Alexa Fluor 594-conjugated secondary. Nuclei were stained with DAPI. Scale bar, 20 µm. **g** Immunoblot analysis of C-HGF and actin expression in normal brain (NB) and primary GBM tumor samples

*Circ-HGF* RNA junction reads in ribosome profiling experiments were detected in all four primary GBM tumor samples tested and not found in any of the normal brain samples (Fig. 2A, *top*). We identified a potential IRES within the *circ-HGF* RNA 211 nucleotides upstream of the cross-junction within the RNA circle (Fig. 2A, *bottom*). This IRES was validated in reporter assays utilizing a dual luciferase-split nanoluciferase construct in which nanoluciferase expression and activity is dependent on an IRES driving nanoluciferase translation [18] (Fig. 2B). A firefly luciferase internal control gene also measures cap-dependent initiation, thus both forms of initiation can be ascertained from the same reporter mRNA. Several deletion mutants of the native IRES were constructed and as shown in Fig. 2C, the full-length IRES (47 nucleotides) was required for activity. A cross-junction open reading frame driven from this IRES potentially encoded a 119-amino acid protein (C-HGF) (see Fig. 2A, Online Resource 4 Suppl. Fig. S3). As C-HGF possesses a unique 49 amino acid terminal sequence formed by a ribosomal frameshift occurring at the circular RNA junction, we generated a specific antibody to this unique sequence and the antibody detected a specific protein at 13.6 kDa in GBM6 and HK296 cells (Fig. 2D). We confirmed the expression of C-HGF via mass spectroscopy analysis of immunoprecipitated protein using this antibody (Fig. 2E & Online Resource 5 Suppl. Table 1). C-HGF expression was also detectable in primary GBM via immunofluorescence microscopy analysis (Fig. 2F). Moreover, elevated C-HGF was expressed in primary GBM samples relative to normal brain as determined via immunoblotting utilizing the C-terminal 49 amino acid-specific antibody (Fig. 2G). Taken together these data demonstrate that *circ-HGF* encodes a 119 amino acid protein whose translation is mediated via an IRES and is highly expressed in GBM.

### C-HGF is a secretory protein, activates STAT3, AKT and ERK signaling and regulates GBM properties

**Fig. 3:**
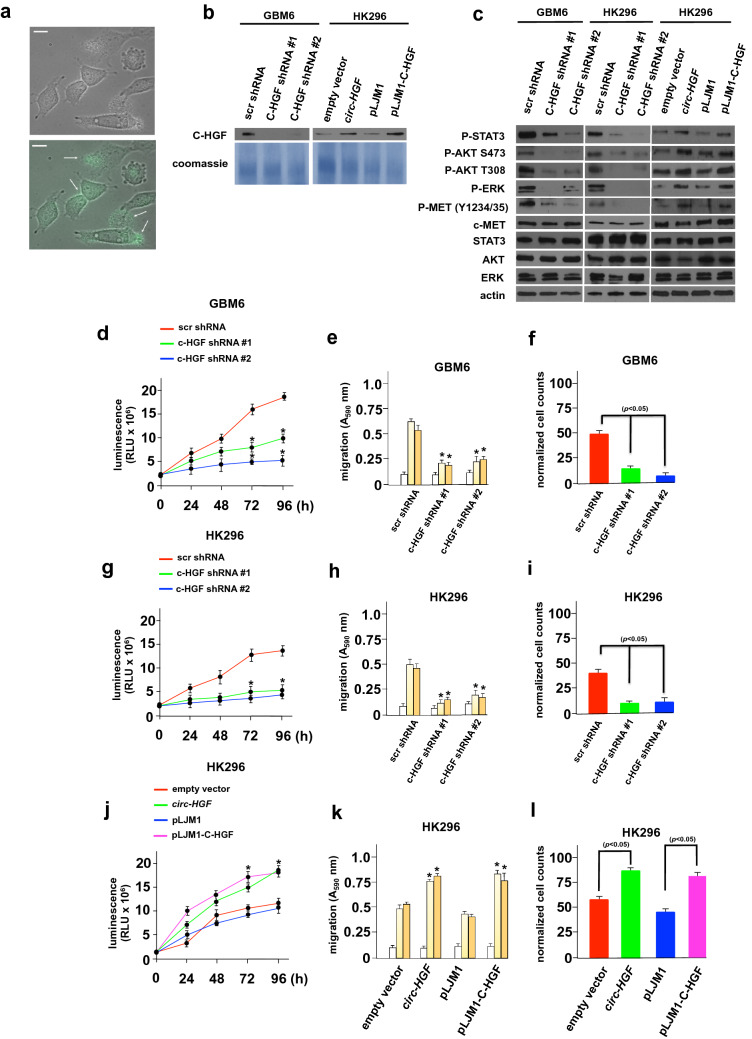
C-HGF is secreted and activates STAT3, AKT and MEK signaling pathways regulating growth, migration and invasive characteristics. **a** Live-cell fluorescence images of HK296 cells transduced with C-HGF-GFP. The arrows indicate secretory C-HGF. Scale bar, 20 µm. **b** Immunoblot of concentrated culture supernatant from GBM6 and HK296 transduced with the indicated constructs (GBM6; nontargeting scramble shRNA sequence, scr shRNA; C-HGF targeting shRNA #1 & #2; HK296; empty RNA expression vector (pLKO.1), RNA expression vector overexpressing *circ-HGF*, pLJM1 control, pLJM1-C-HGF expression construct). Coomassie blue staining of total protein was used as a lane loading control. **c** Expression of the indicated c-MET effectors in GBM6 or HK296 cells stably transduced with the indicated constructs. **d** Effects of C-HGF knockdown on GBM6 cell growth. ATP-release assays (Promega CellTiter-Glo®) were used to quantify growth and displayed in relative light units of the indicated cell lines. Mean + S.D. are shown; *n* = 3; *, *p* < 0.05. **e** C-HGF knockdown inhibits GBM6 migration in C-HGF knockdown cells. The indicated modified lines were placed in Boyden chambers and allowed to migrate towards BSA (white bars), vitronectin (light yellow bars), or fibronectin (dark yellow bars). Mean + S.D., *, *p* < 0.05, n = 3. **f** Invasive potential of the indicated GBM6 knockdown lines migrating through Matrigel. Data represent mean + S.D. of three independent experiments. Effects of *circ-HGF* RNA or C-HGF ORF overexpression in stably transduced HK296 cells on proliferation (**g**), migration (**h**) and invasiveness (**i**). As in **d**-**f**, respectively

To assess whether C-HGF displayed secretory properties and may stimulate c-Met signaling, we initially generated a GFP-tagged version of C-HGF and expressed this protein in HK296 cells. As shown in Fig. 3A, C-HGF-GFP was soluble and was secreted from cells. To examine whether supernatants from cells in which we modulated C-HGF expression would secrete the active protein, we generated stable knockdowns of C-HGF via transduction of shRNAs targeting C-HGF in GBM6 and HK296 cells. Knockdown of C-HGF was specific in that expression of endogenous native HGF in these cells was unaffected (Online Resource 6 Suppl. Fig. S4). HK296 cells were stably transduced with either a construct expressing *circ-HGF* or stably transduced with a lentiviral expression vector into which the ORF for C-HGF had been inserted. Supernatants from these cells were concentrated and immunoblotted for C-HGF (Fig. 3B). These cells were also subsequently analyzed by immunoblot for the activities of known c-MET effectors (Fig. 3C). Cells in which C-HGF had been knocked down displayed markedly reduced P-STAT3, P-S^437^-AKT, P-T^308^-AKT, and P-ERK levels, while in cells overexpressing C-HGF these substrates were significantly elevated. c-MET phosphorylation was also inhibited in the C-HGF knockdown lines and displayed increased levels in the overexpressing lines. These data demonstrated that C-HGF was capable of activating the STAT3, AKT and MAPK pathways, known effectors of c-MET signaling. To examine the effects of modulating C-HGF expression on growth, migratory capacity and invasiveness we assessed these properties in the PDX GBM lines. As shown in Fig. 3D-F, GBM6 C-HGF knockdown lines exhibited inhibition of growth, migration and invasive characteristics as compared to cells transduced with a nontargeting control shRNA. In contrast, HK296 cells stably overexpressing either the *circ-HGF* RNA or C-HGF protein displayed marked increases in growth, migration and invasion relative to control cells (Fig. 3G-I). These results demonstrate that C-HGF is secreted from cells regulating STAT3, AKT and ERK signaling, as well as GBM cell properties.

### C-HGF/c-MET signaling controls GBM cell growth, migration and invasion

**Fig. 4:**
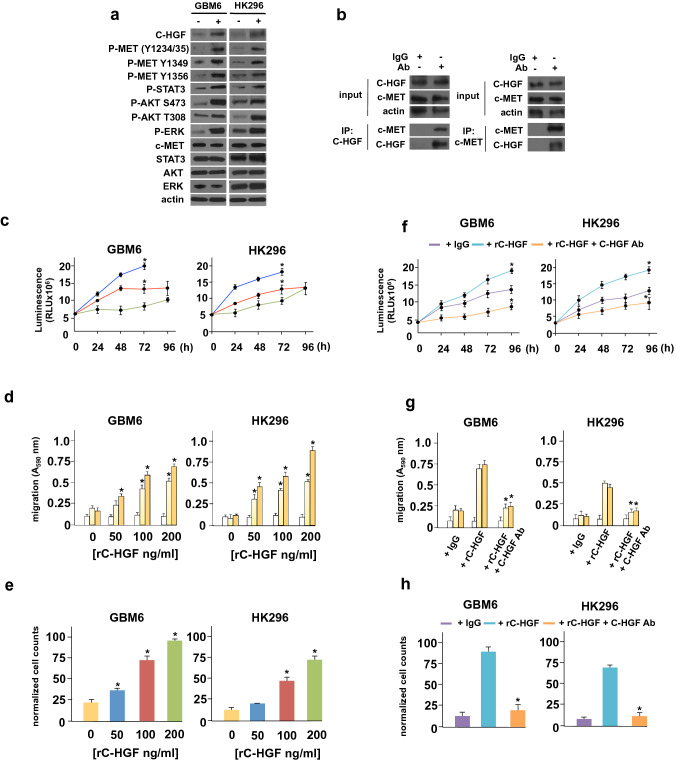
C-HGF exhibits direct effects on c-MET activity regulating GBM properties. **a** Effects of recombinant C-HGF (rC-HGF) on c-MET phosphorylation and downstream effector signaling in GBM6 and HK296 GBM cells. Cells were treated with rC-HGF (200 ng/ml) for 8 h and subsequently immunoblotted for the indicated proteins. **b** C-HGF co-immunoprecipitates with endogenous c-MET in GBM6 cells. GBM6 cell extracts were immunoprecipitated with control IgG, α-C-HGF or α-c-MET antibodies as shown and immunoprecipitates probed for the indicated proteins. Input extracts were immunobloted for C-HGF, c-MET and actin. **c** GBM6 or HK296 were treated with either 0 (green), 50 (red) or 200 (blue) ng/ml of rC-HGF and proliferation assessed in ATP-release assays at the indicated timepoints. Mean ± S.D., *, *p* < 0.05, *n* = 3. **d** GBM6 or HK296 cells were placed in Boyden chambers in the presence of the indicated concentrations of rC-HGF and allowed to migrate towards BSA (white bars), vitronectin (light yellow bars) or fibronectin (dark yellow bars). Mean + S.D., *, *p* < 0.05, *n* = 3. **e** Invasive capacity of GBM6 or HK296 cells treated with the indicated concentrations of rC-HGF migrating through Matrigel. Mean + S.D., *, *p* < 0.05, *n* = 3. **f** ATP-release assay analysis of GBM6 or HK296 cells treated with control IgG, rC-HGF (200 ng/ml) or rC-HGF (200 ng/ml) + C-HGF antibody (1 µM) at the indicated timepoints. Mean + S.D., *, *p* < 0.05, *n* = 3. **g** Migration of GBM6 or HK296 towards BSA (white bars), vitronectin (light yellow bars), or fibronectin (dark yellow bars) with the indicated treatments; control IgG, rC-HGF (200 ng/ml) or rC-HGF (200 ng/ml) + C-HGF antibody (1 µM). **h** Invasive potential of GBM6 or HK296 cells with the indicated treatment (control IgG, rC-HGF (200 ng/ml) or rC-HGF (200 ng/ml) + C-HGF antibody (1 µM)) migrating through Matrigel. Mean + S.D., *, *p* < 0.05, *n* = 3

To determine whether C-HGF had direct effects on c-MET activity in GBM cells we generated recombinant His-tagged C-HGF and found that GBM6 or HK296 GBM cells when treated with this recombinant protein (rC-HGF) resulted in significant activation of c-MET as monitored by P-Y^1234^-MET and P-Y^1235^-MET autophosphorylation (Fig. 4A). Additionally, rC-HGF exposure resulted in phosphorylation of c-MET Y^1349^ and Y^1356^ residues that are essential for the recruitment of adaptor proteins involved in c-MET signaling [19–21]. Moreover, we determined whether C-HGF could directly interact with c-MET in HK296 cells and in co-immunoprecipitation experiments, antibodies specific for C-HGF effectively co-immunoprecipitated c-MET (Fig. 4B). Co-immunoprecipitating in the reciprocal fashion with c-MET antibodies, C-HGF was also detectable in c-MET immunoprecipitates. We subsequently examined the effects of rC-HGF on GBM6 and HK296 cell proliferation, migration and invasive capacity. As shown in Fig. 4C, treatment with rC-HGF increased the proliferation of both PDX lines in a dose-dependent fashion. Similarly, increases in cell mobility and invasiveness were observed in these lines upon exposure to rC-HGF (Fig. 4D-E). We then determined whether the C-HGF specific antibody targeting the c-terminal unique sequence would neutralize the stimulatory effects of rC-HGF exposure in GBM6 and HK296 cells. As shown in Figs. 4F-H, co-addition of C-HGF antibody to rC-HGF treated cultures markedly reduced proliferation, migration and invasiveness of these PDX GBM lines compared to controls. We also evaluated the responses of GBM6 and HK296 cells in which c-MET expression was stably knocked down via shRNAs to rC-HGF. As shown in Supplementary Figure S5A (Online Resource 7), treatment of cells with rC-HGF induced c-MET signaling in control non-targeting scr shRNA expressing cells and was blunted or undetectable in c-MET targeting shRNA expressing cells. While c-MET knockdown significantly reduced GBM6 and HK296 growth, motility and invasiveness relative to scr shRNA expressing control cells, c-MET knockdown GBM6 and HK296 cells displayed no significant increases in growth, motility or invasive character in response to rC-HGF as compared to the marked induction observed in control scr shRNA expressing cells (Supplementary Figure S5B-D). Taken together these data suggest that C-HGF's effects are mediated via c-MET.

### Effects of C-HGF modulation on in vivo GBM growth

**Fig. 5:**
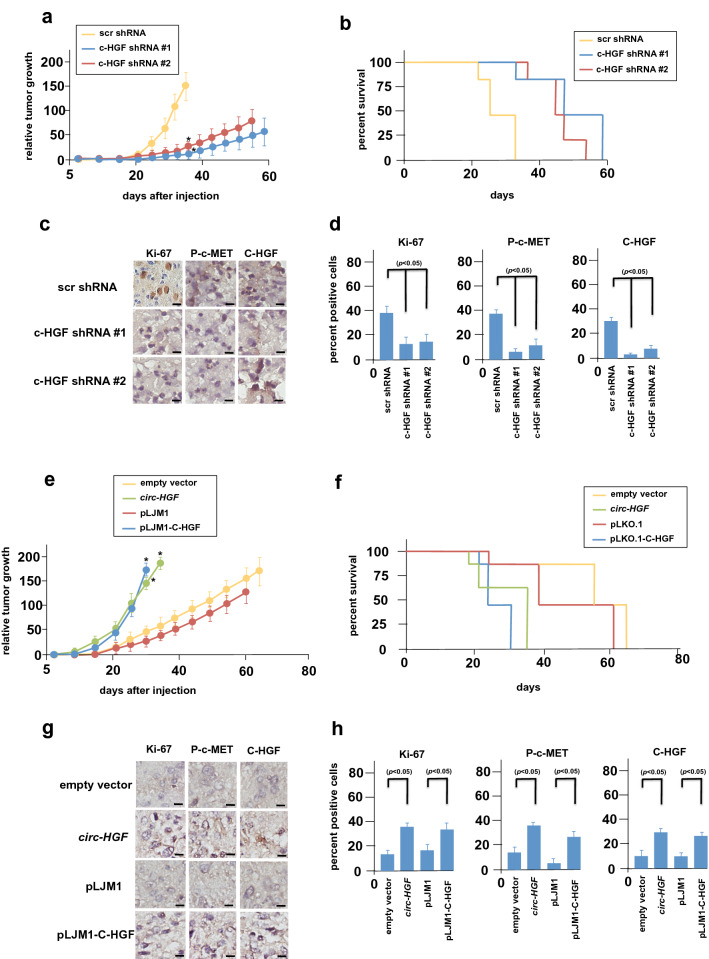
In vivo growth of C-HGF knockdown or overexpressing PDX lines. **a** Athymic nude mice were injected intracranially with 1 × 10^6^ luciferase-tagged GBM6 cells expressing the indicated control or knockdown shRNA lines (shRNA #1 & #2) and tumor burden measured by bioluminescence on the indicated days post-injection (*n* = 6 per group). *, *p* < 0.05. **b** Kaplan–Meier analysis of survival data of the indicated xenografted mice (*n* = 6 per group). **c** Harvested tumors were sectioned (day 38 post-injection) and subjected to immunohistochemical staining for Ki-67, p-Y^1234/1235^-c-MET and C-HGF expression. Scale bar, 20 µm. **d** Percentage of Ki-67, p-Y^1234/1235^-c-MET and C-HGF immunopositive (brown) cells in GBM6 xenograft tumors. **e** Growth of intracranial xenographs of mice injected with 1 × 10^6^ luciferase tagged HK296 cells expressing the indicated constructs. Tumor burden was measured by bioluminescence as in (**a**). *, *p* < 0.05, *n* = 6 per group. **f** Kaplan-Meir survival analysis of mice bearing the indicated HK296 cell line xenografted tumors (*n* = 6 per group). **g** Immunohistochemical analysis of Ki-67, p-Y^1234/1235^-c-MET and C-HGF in the indicated HK296 cell line xenografted tumors (day 30 post-injection). Scale bar, 20 µm. **h** Percentage of Ki-67, p-Y^1234/1235^-c-MET and C-HGF positive cells in HK296 xenograft tumors

To examine the effects of C-HGF on in vivo tumor growth we utilized the C-HGF knockdown GBM6 and the C-HGF overexpressor HK296 PDX lines we previously generated (see Fig. 3). Intracranial xenografts of luciferase-tagged GBM6 cells expressing a non-targeting shRNA and two independent C-HGF targeting shRNA lines (C-HGF shRNA #1 & 2) were established and effects on in vivo growth determined. As shown in Fig. 5A, knockdown of C-HGF markedly inhibited the growth and delayed the onset of tumor progression as compared to xenografts expressing the non-targeting control shRNA (scr shRNA tumor onset = day 22; shRNAs #1 & #2 tumor onset = day 32). Mice bearing the C-HGF targeting shRNA xenografts also displayed a significant increase in overall survival relative to the control non-targeting shRNA group (Fig. 5B). Harvested tumors were sectioned and immunohistochemically stained for Ki-67, phospho-c-MET and C-HGF expression (Fig. 5C). As shown in Fig. 5d, knockdown of C-HGF significantly reduced the percentage of positive cells expressing Ki-67, phospho-c-MET and C-HGF as compared with control non-targeting shRNA expressing GBM6 cells. In mice harboring intracranial xenografts of HK296 cells stably overexpressing *circ-HGF* RNA or the C-HGF ORF, these xenografts displayed increased tumor growth and shortened the time of tumor onset (empty vector & pLKO.1, tumor onset = 21 days; *circ-HGF*, tumor onset = day 8; pLKO.1-C-HGF, tumor onset = day 10) relative to controls (Fig. 5E). Overall survival of mice with tumors overexpressing *circ-HGF* RNA or the C-HGF ORF was also significantly reduced as compared to controls (Fig. 5F). Ki-67, phospho-c-MET and C-HGF expression was markedly higher in *circ-HGF* and C-HGF ORF tumors than controls consistent with the increase in observed growth (Fig. 5G, H).

## Discussion

Significant attention has been recently focused on the translation of circRNAs to determine their possible clinical relevance in GBM [14]. Recent reports have described novel protein isoforms translated from circRNAs via IRES-mediated protein synthesis which are distinct from their linear mRNA counterparts [22–25]. In the current study we identified a 119 aa variant of HGF whose translation is mediated by an IRES element within the *circ-HGF* RNA. The translation product, C-HGF, is secreted by GBM cells and is capable of stimulating c-MET signal cascade activity. Our data also suggests that C-HGF directly binds to c-MET resulting in its direct autophosphorylation and activation, as well as stimulating c-MET downstream effectors. shRNA-mediated blockade of C-HGF expression in PDX GBM lines resulted in marked inhibition of GBM growth, migration and invasive properties, while overexpression of C-HGF conferred the opposite effects in vitro. Recombinant C-HGF stimulated growth and associated GBM properties which were inhibited by co-treatment with a neutralizing antibody targeting a unique 49 residue c-terminal sequence within C-HGF. Knockdown or overexpression of C-HGF expression in PDX lines recapitulated the effects on GBM cell properties observed in vitro, in terms of tumor burden and overall survival in xenograft studies in mice.

Our data are consistent with a model in which dysregulation of C-HGF expression in GBM results in activation of c-MET signaling promoting downstream signaling driving growth, motility and invasiveness in GBM in a paracrine/autocrine fashion. This is supported by our observations that C-HGF is secreted from GBM cells and can co-immunoprecipitate with c-MET leading to autophosphorylation of the receptor (see Figs. 2A&B, 3B). Moreover, the observation that c-MET phospho-Y^1349^ and phospho-Y^1356^ levels are enhanced following rC-HGF stimulation suggest that the docking platform which serves to recruit additional c-MET interactors such as Gab1, STAT3 and Ras is functional (see Fig. 4A) [19–21]. STAT3 is known to associate with phosphorylated c-MET and undergo phosphorylation itself, which was also seen in our experiments following overexpression or exposure to rC-HGF (Figs. 3C& 4A) [26–28]. These data suggest that C-HGF may be competent to activate c-MET in an analogous manner as its natural ligand. It will be of interest to determine whether c-MET endocytosis and recycling are differentially affected via engagement by the two ligands, as this mechanism tightly regulates sustained c-MET activation [29].

The IRES-*trans*-acting factor (ITAF) requirements for IRES-mediated translation of circRNAs have not been explored in depth. The expression of mRNAs bearing IRES elements is controlled by multiple mechanisms and enhanced when canonical cap-dependent initiation is compromised [30]. Additionally, it has been demonstrated that an IRES within a particular mRNA can respond differently to various conditions which inhibit cap-dependent translation depending on particular ITAF-IRES interactions [30–32]. While it seems likely that ITAFs, which regulate IRES activity in mRNAs, will have similar affects on IRES activity found in circRNAs, this remains to be confirmed. Future studies aimed at identifying and characterizing the relevant ITAFs mediating circRNA IRES-dependent initiation are warranted.

Translation on circRNAs can also be initiated as a result of m^6^A modification within DRACH motifs [33]. These sequences containing m^6^A-induced ribosome engagement sites (MIRESs) have been reported to function as IRESs to drive circRNA translation [34, 35]. m^6^A modifications can be enriched in circRNAs and a single m^6^A may be sufficient to initiate translation. Furthermore, circRNA translation has been reported to be stimulated by overexpression of the major methyltransferase complex METTL3/4 and inhibited by the m^6^A demethylase FTO [35]. It has also been suggested that some degree of cooperation may exist between IRES and m^6^A-dependent circRNA translation initiation [12]. We identified several consensus DRACH motifs within *circ-HGF*, however we were unable to detect m^6^A methylation of *circ-HGF* at these motifs via anti-m^6^A antibody immunoprecipitation and subsequent qRT-PCR analysis in GBM PDX lines (not shown). This suggests that C-HGFs primary mechanism of translation initiation is IRES-dependent.

In conclusion, these studies identified a novel *circ-HGF* derived protein variant of HGF which is secreted by GBM cells and stimulates c-MET signaling leading to enhanced growth, motility and invasive characteristics. C-HGF was found to be highly expressed in GBM patient samples and promoted PDX cell growth In vitro and in xenografts. C-HGF was found to be translated via an IRES-dependent mechanism and contains 49 unique c-terminal residues which may serve as an effective anticancer target.

## Supplementary Information

Below is the link to the electronic supplementary material.

Supplementary file1 (PDF 124 KB)

Supplementary file2 (PDF 82 KB)

Supplementary file3 (PDF 99 KB)

Supplementary file4 (PDF 191 KB)

Supplementary file5 (XLSX 82 KB)

Supplementary file6 (PDF 101 KB)

Supplementary file7 (PDF 268 KB)

## Data Availability

The datasets generated during the course of this study are available from the corresponding author upon reasonable request.
