# Supplementary material for: Translation of circHGF RNA encodes an HGF protein variant promoting glioblastoma growth through stimulation of c-MET
Source: J Neurooncol. 2023 May 10;163(1):207–18. doi: 10.1007/s11060-023-04331-5 (PMC10232650; doi:10.1007/s11060-023-04331-5)
Supplement: Supplementary file 1 — Supplementary file1 (PDF 124 KB) [file 11060_2023_4331_MOESM1_ESM.pdf]

Online Resource 1. Supplemental Materials and Methods

Translation of circHGF RNA encodes an HGF protein variant promoting glioblastoma growth through stimulation of c-MET

Authors and Affiliations

Jacquelyn T. Saunders[1,5], Sunil Kumar[5], Angelica Benavides-Serrato[5], Brent Holmes[5], Kennedy E. Benavides[5], Muhammad T. Bashir[5], Robert N. Nishimura[2,5] and Joseph Gera[1,3,4,5]

Department of [1]Medicine and [2]Neurology, David Geffen School of Medicine at UCLA, [3]Jonnson Comprehensive Cancer Center, [4]Molecular Biology Institute, University of California-Los Angeles, California and [5]Department of Research & Development, Greater Los Angeles Veterans Affairs Healthcare System, Los Angeles, California

Corresponding Author: Joseph Gera, Ph.D., Greater Los Angeles VA Healthcare System, 16111 Plummer Street (151), Building 1, Room C111A, Los Angeles, CA 91343. Phone: (818) 895-9416; Fax: (818) 895-9554; E-Mail: jgera@mednet.ucla.edu

Cell Lines, primary GBM samples, retroviral transduction and DNA transfection

Short-term PDX lines were kindly provided by Dr. Jann Sarkaria (PDX National Resource, Translational Neuro-Oncology, Mayo Clinic) and Dr. Harley Kornblum (Department of Molecular and Medical Pharmacology, UCLA). Normal mature human neurons were obtained from ScienCell (Carlsbad, CA). Flash-frozen normal brain and glioblastoma samples were obtained from the Cooperative Human Tissue Network, National Cancer Institute (Western Division, Vanderbilt University Medical Center) under an institutional review board approved protocol. Each glioblastoma sample was histopathologically reviewed and those containing greater than 95% tumor were utilized in this analysis. Lentiviral constructs were introduced into EcoPack2-293 cells and tittered as described by the manufacturer (Clontech). Prior to infection, GBM6 or HK296 cells were grown to 80% confluency, pretreated with 6 mg/ml polybrene, and then infected with a multiplicity of infection of ~1.5-2. DNA transfections were performed using Effectene transfection reagent according to the manufacturer (Qiagen).

Constructs and reagents

The circular RNA IRES reporter construct, pcDNA3.1 ZKSCAN split nanoluciferase with the firefly luciferase internal control, has been described previously [1] (generously provided by Dr. Gabriele Fuchs, Department of Biological Sciences, The RNA Institute, University of Albany, SUNY) and the full-length *wt* circ-HGF IRES, the indicated truncated mutants (Fig. 2B), as well as human CCND1 IRES sequences [2] were cloned into the MCS. The shRNA constructs (C-HGF shRNA #1 & #2 and c-MET shRNA) targeting *circ-HGF* cross-junctional spanning sequences within the coding ORFs of exon 6 and exon 11 or sequences within the coding region of c-MET, respectively, and the scrambled (scr shRNA) sequence construct (pLKO.1, plasmid #1864, Addgene) were pLKO.1-based. The full-length C-HGF ORF was cloned into pCDH-CMV-MCS-EF1-Puro (SBI, Palo Alto, CA) and pLJM1 to express *circ-HGF* RNA and C-HGF, respectively. The C-HGF-GFP expression construct was generated by cloning the ORF fused to green fluorescent protein GFP). Recombinant C-HGF was purified from HEK293 cells using Sepharose column chromatography as previously described [3]. Antibodies to the following proteins were used: phospho-S^{473}-AKT (CST, #9271), phospho-T^{308}-AKT (CST, #9275), AKT (CST, #9272), phospho-Y^{705}-STAT3 (CST, #9145), STAT3 (CST, #9139), phospho-T^{202}/Y^{204}-ERK (CST, #4377), ERK (ThermoFisher, #13-6200), actin (abcam, #ab3280), c-MET (abcam, #ab51067), phospho-Y$^{1234/35}$-MET (CST, #3126), phospho-Y^{1349}-MET (CST, #3121), phospho-Y^{1356}-MET (Rockland, #600-401-989) and Ki-67 (CST, #9449). The C-HGF antibody was generated in rabbits immunized with the peptide ARFVSAGIIRHHTGTNS and subsequently affinity purified [4]. Antibody blocking experiments utilized this peptide. RNase-R (MCLAB, San Francisco, CA) treatment was performed by adding 2 units per microgram of RNA and samples incubated for 1 h at 37˚ C.

RNAseq and ribosome profiling sequencing

Total RNA from GBM or normal brain samples was isolated using Trizol reagent (Invitrogen). Following rRNA depletion, purification and reverse transcription the products were subjected to Illumina sequencing. Putative circRNAs were counted if the unique junction reads were 2 or greater. CircRNAs

with a fold change of 2.5 or greater ($p < 0.05$) were considered differentially expressed circRNAs. Clean reads were mapped to a reference genome using the STAR algorithm. Differential transcript abundance was determined using DESeq2. Ribosomal profiling was performed as previously described [5]. Libraries were constructed using the NEBNext Multiple Small RNA Library Prep Set for Illumina according to the manufacturer (New England Biolabs). Briefly, adapters were added to fragments, reverse transcribed and products amplified to generate cDNA libraries and sequenced using the Illumina HiSeq platform. Poor quality reads were filtered using FASTP and the remaining reads were mapped to a reference genome using Bowtie2. CircRNA data was obtained from circBase (http://www.circbase.org).

Immunoblotting, Northern blotting and quantitative real time PCR

Western blotting was performed as previously described [6]. Total RNA was reverse transcribed into cDNA using the High Capacity RNA-to-cDNA kit (ABI). Taqman primers (from Applied Biosystems) were used to measure the levels of all transcripts and analyses were performed on an ABI7900HT system (ABI ThermoFisher). Northern blotting was performed as described [7].

Mass spectrometry analysis

Following immunoprecipitation utilizing the C-HGF specific antibody samples were subjected to SDS-PAGE and in-gel digested with trypsin. Samples were analyzed on a LTQ Velos Dual-Pressure Ion Trap Mass Spectrometer (Thermo Scientific) and protein fragments identified using MASCOT.

Cell fractionation, immunoprecipitation and IRES reporter assays

Nuclear-cytoplasmic fractionation was performed according to Dignam et al. [8]. Briefly, all buffers used were kept on ice and centrifugations were done at 4 °C with soft braking. After a single wash with PBS, cells were scraped with PBS (containing 1 mM DTT and 1× protease inhibitor) and harvested by centrifugation at 1000 × g for 15 min. The cell pellet was gently resuspended with five times the volume of pellet with buffer A (10 mM HEPES, pH 7.9, 1.5 mM $MgCl_2$, 10 mM KCl, 0.5 mM DTT, and

1× protease inhibitor) and incubated on ice for 15 min, followed by homogenization. The cell lysate was spun at 1000 × g for 5 min to collect the pellet as the nuclear fraction and the supernatant as the cytoplasmic fraction. Immunoprecipitations were performed as previously described (6). For IRES reporter assays, the indicated reporters were cotransfected into cells with pSVβ-galactosidase to normalize for transfection efficiency as described previously [2]. Cells were harvested 18 h following transfection and firefly and NanoLuc activities were measured in a Glomax instrument using the Dual Nano-Glo kit (Promega).

Immunofluorescence and immunohistochemistry

For immunofluorescence staining, cells were grown on coverslips and were fixed with 4% paraformaldehyde in PBS for 15 min at room temperature (or overnight at 4˚C) and washed three times for 5 min in 100 mM glycine containing PBS, followed by permeabilization with 0.1% Triton X-100 in PBS for 10 min. After blocking with 3% nonfat dry milk in PBS for 1 h, cells were incubated with primary antibody diluted in 1% BSA/PBS overnight at 4˚C. After washing with PBS, cells were incubated with Alexa Fluor 594–conjugated secondary antibodies (Invitrogen) for 1 h and washed with PBS. Cell nuclei were counterstained and mounted with a mounting medium with DAPI (Vectashield; Vector Laboratories). Immunofluorescence staining images were collected at room temperature on a Zeiss Axio Imager M2 microscope coupled to a cooled digital CCD camera (ORCA®-R^2 C10600-10B-H; Hamamatsu Photonics). Immunohistochemical analysis was performed according to standard procedures with antigen retrieval performed as previously described [9].

Cell proliferation, migration and invasion assays

Cell proliferation was determined via Cell Titer-Glo® luminescent cell assays as described by the manufacturer (Promega). Cell migration assays were conducted using precoated modified Boyden chambers as previously described [10,11]. For invasion assays through Matrigel®, 20,000 cells were loaded in the top well of Boyden chambers that contained growth factor-reduced Matrigel® extracellular

basement membrane over a polyethylene terephthalate membrane with 8-mm pores (BD Biosciences) [10,11]. Migration and invasion data were normalized to total numbers of cells.

Xenograft studies

All experiments were performed under an approved Institutional Animal Care and Use Committee protocol (Greater Los Angeles VA Healthcare System, protocol # 01002-14) and conformed to the guidelines established by the Association for the Assessment and Accreditation of Laboratory Animal Care. Intracranial xenografts of the indicated PDX cells were established in 4-6-week-old female athymic NCr nude mice (Taconic) as previously described [12]. For bioluminescence imaging, mice were injected intraperitoneally with d-luciferin, imaged utilizing an IVIS *in vivo* imaging system and photonic emissions assessed. Tumors were harvested at autopsy and sections of paraffin-embedded tumors on slides were processed for immunohistochemistry as previously described [13].

Statistical analysis

Statistical analyses were performed using unpaired Student's t tests and ANOVA models using Systat 13 (Systat Software, Chicago, IL) along with a Dunnett's test unless otherwise described. P values of less then 0.05 were considered significant.

References for Supplemental Materials and Methods

1. Sehta P, Wilhelm AM, Lin SJ, Urman MA, MacNeil HA, Fuchs G. (2002) A Split NanoLuc Reporter Quantitatively Measures Circular RNA IRES Translation. Genes (Basel). 13(2):357. doi: 10.3390/genes13020357.
2. Holmes B, Lee J, Landon KA, Benavides-Serrato A, Bashir T, Jung ME, Lichtenstein A, Gera J. (2016) Mechanistic Target of Rapamycin (mTOR) Inhibition Synergizes with Reduced Internal Ribosome Entry Site (IRES)-mediated Translation of Cyclin D1 and c-MYC mRNAs to Treat Glioblastoma. J Biol Chem. 291(27):14146-14159. doi: 10.1074/jbc.M116.726927.
3. Urh M, Simpson D, Zhao K. (2009) Affinity chromatography: general methods. Methods Enzymol. 463:417-38. doi: 10.1016/S0076-6879(09)63026-3.
4. Jo OD, Martin J, Bernath A, Masri J, Lichtenstein A, Gera J. (2008) Heterogeneous nuclear ribonucleoprotein A1 regulates cyclin D1 and c-myc internal ribosome entry site function through Akt signaling. J Biol Chem. 283(34):23274-87. doi: 10.1074/jbc.M801185200.

5. Ingolia NT, Brar GA, Rouskin S, McGeachy AM, Weissman JS. (2012) The ribosome profiling strategy for monitoring translation in vivo by deep sequencing of ribosome-protected mRNA fragments. Nat Protoc. 7(8):1534-50. doi: 10.1038/nprot.2012.086.
6. Artinian N, Cloninger C, Holmes B, Benavides-Serrato A, Bashir T, Gera J. (2015) Phosphorylation of the Hippo Pathway Component AMOTL2 by the mTORC2 Kinase Promotes YAP Signaling, Resulting in Enhanced Glioblastoma Growth and Invasiveness. J Biol Chem. 290(32):19387-401. doi: 10.1074/jbc.M115.656587.
7. Gera JF, Mellinghoff IK, Shi Y, Rettig MB, Tran C, Hsu JH, Sawyers CL, Lichtenstein AK. (2004) AKT activity determines sensitivity to mammalian target of rapamycin (mTOR) inhibitors by regulating cyclin D1 and c-myc expression. J Biol Chem. 279(4):2737-46. doi: 10.1074/jbc.M309999200.
8. Dignam JD, Lebovitz RM, Roeder RG. (1983) Accurate transcription initiation by RNA polymerase II in a soluble extract from isolated mammalian nuclei. Nucleic Acids Res. 11(5):1475-89. doi: 10.1093/nar/11.5.1475.
9. Bashir T, Cloninger C, Artinian N, Anderson L, Bernath A, Holmes B, Benavides-Serrato A, Sabha N, Nishimura RN, Guha A, Gera J. (2012) Conditional astroglial Rictor overexpression induces malignant glioma in mice. PLoS One. 7(10):e47741. doi: 10.1371/journal.pone.0047741.
10. Holmes B, Benavides-Serrato A, Saunders JT, Kumar S, Nishimura RN, Gera J. (2021) mTORC2-mediated direct phosphorylation regulates YAP activity promoting glioblastoma growth and invasive characteristics. Neoplasia. 23(9):951-965. doi: 10.1016/j.neo.2021.07.005.
11. Pijuan J, Barceló C, Moreno DF, Maiques O, Sisó P, Marti RM, Macià A, Panosa A. In vitro Cell Migration, Invasion, and Adhesion Assays: From Cell Imaging to Data Analysis. Front Cell Dev Biol. 2019 Jun 14;7:107. doi: 10.3389/fcell.2019.00107.
12. Bi J, Khan A, Tang J, Armando AM, Wu S, Zhang W, Gimple RC, Reed A, Jing H, Koga T, Wong IT, Gu Y, Miki S, Yang H, Prager B, Curtis EJ, Wainwright DA, Furnari FB, Rich JN, Cloughesy TF, Kornblum HI, Quehenberger O, Rzhetsky A, Cravatt BF, Mischel PS. (2021) Targeting glioblastoma signaling and metabolism with a re-purposed brain-penetrant drug. Cell Rep. 37(5):109957. doi: 10.1016/j.celrep.2021.109957.
13. Garner JM, Ellison DW, Finkelstein D, Ganguly D, Du Z, Sims M, Yang CH, Interiano RB, Davidoff AM, Pfeffer LM. (2015) Molecular heterogeneity in a patient-derived glioblastoma xenoline is regulated by different cancer stem cell populations. PLoS One. 10(5):e0125838. doi:10.1371/journal.pone.0125838.
