# Supplementary material for: Translation of circHGF RNA encodes an HGF protein variant promoting glioblastoma growth through stimulation of c-MET
Source: J Neurooncol. 2023 May 10;163(1):207–18. doi: 10.1007/s11060-023-04331-5 (PMC10232650; doi:10.1007/s11060-023-04331-5)
Supplement: Supplementary file 2 — Supplementary file2 (PDF 82 KB) [file 11060_2023_4331_MOESM2_ESM.pdf]

		RPF count	
gene	circBase RNA ID	NB	GBM
circ-HGF	*hsa_circ_0080914*	0	11
circ-E-Cad	*hsa_circ_0039992*	0	8
circ-Rictor	*hsa_circ_0129052*	3	5
circ-PINK1	*hsa_circ_0111789*	1	7
circ-TAZ	*hsa_circ_0139927*	3	9
circ-Rictor	*hsa_circ_0129052*	3	7
circ-PTPRZ1	*hsa_circ_0133169*	6	11
circ-RALY	*hsa_circ_0114956*	12	4
circ-KIT	*hsa_circ_0126617*	5	6
circ-CNST	*hsa_circ_0112857*	13	2
circ-PAX6	*hsa_circ_0095689*	3	9
circ-GLI3	*hsa_circ_0134316*	4	7

Supplementary Figure S1. Twelve differentially expressed coding circRNAs which were annotated in circBase and numbers of ribosome protected fragments (RFPs) detected in GBM and normal brain (NB).
