# Supplementary material for: Translation of circHGF RNA encodes an HGF protein variant promoting glioblastoma growth through stimulation of c-MET
Source: J Neurooncol. 2023 May 10;163(1):207–18. doi: 10.1007/s11060-023-04331-5 (PMC10232650; doi:10.1007/s11060-023-04331-5)
Supplement: Supplementary file 3 — Supplementary file3 (PDF 99 KB) [file 11060_2023_4331_MOESM3_ESM.pdf]

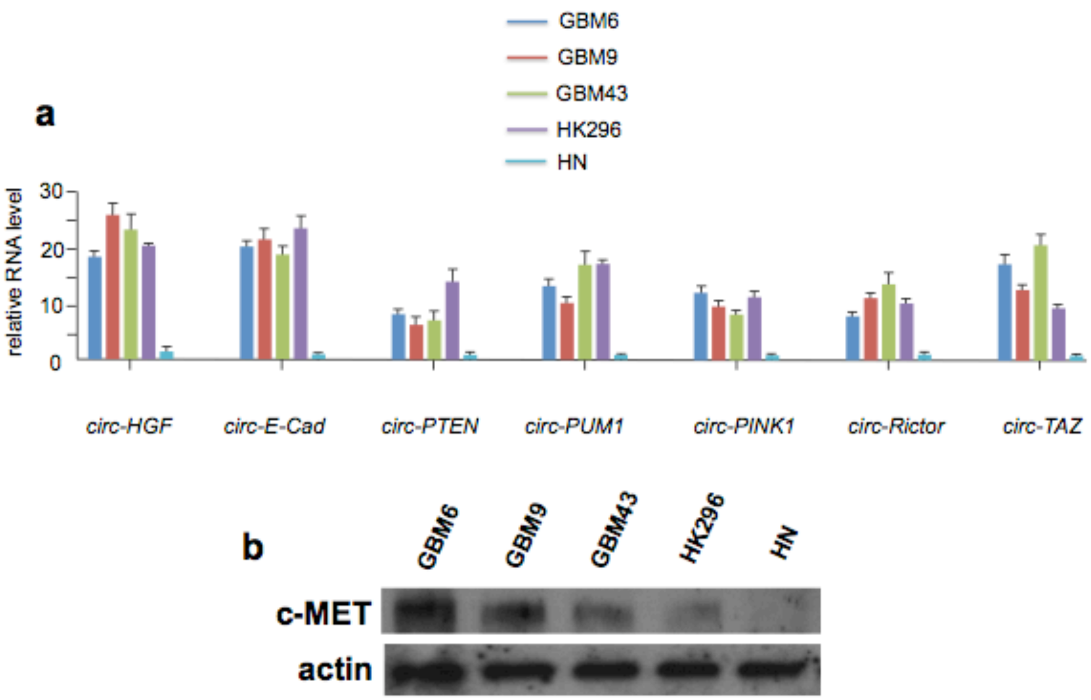

Supplementary Figure S2. a Relative expression levels of the indicated circRNAs as determined via qRT-PCR in the GBM PDX lines shown. + S.D., n = 3. **b** c-MET and actin protein levels in the indicated GBM PDX lines and human neurons (HN).
