# Supplementary material for: Translation of circHGF RNA encodes an HGF protein variant promoting glioblastoma growth through stimulation of c-MET
Source: J Neurooncol. 2023 May 10;163(1):207–18. doi: 10.1007/s11060-023-04331-5 (PMC10232650; doi:10.1007/s11060-023-04331-5)
Supplement: Supplementary file 6 — Supplementary file6 (PDF 101 KB) [file 11060_2023_4331_MOESM6_ESM.pdf]

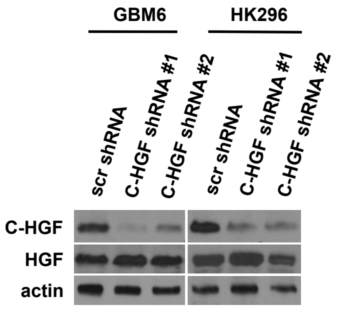

Supplementary Figure S4. C-HGF and endogenous HGF protein levels in GBM6 and HK296 cells expressing the control nontargeting scramble (scr) sequence or C-HGF targeting shRNAs as indicated.
