# Supplementary material for: Translation of circHGF RNA encodes an HGF protein variant promoting glioblastoma growth through stimulation of c-MET
Source: J Neurooncol. 2023 May 10;163(1):207–18. doi: 10.1007/s11060-023-04331-5 (PMC10232650; doi:10.1007/s11060-023-04331-5)
Supplement: Supplementary file 4 — Supplementary file4 (PDF 191 KB) [file 11060_2023_4331_MOESM4_ESM.pdf]

a

>hsa_circ_0080914
UUGAAUGCAUGGCCUGCAAUGGGGAGAGUUAUCGAGGUCUCAUGGA
UCAUACAGAAUCAGGCAAGAUUUGUCAGCGCUGGGAUCAUCAGACA
CCACACCGGCACAAAUUCUUGCCUGAAAGAUAUCCCGACAAGGGCU
UUGAUGAUAUGAAUUGCCGCAAUCCCGAUGGCCAGCCGAGGCCAUG
GUGCUAUACUCUUGACCCUCACACCCGCUGGGAGUACUGUGCAAUU
AAAACAUGCGCUGACAAUACUAUGAAUGACACUGAUGUUAAUUUGGA
AACAACUGAAUGCAUCCAAGGUCAAGGAGAAGGCUACAGGGGCACU
GUCAAUACCAUUUGGAAUGGAAUUCCAUGUCAGCGUUGGGAUUCUC
AGUAUCCUCACGAGCAUGACAUGACUCCUGAAAUUUCAAGUGCAAG
GACCUACGAGAAAAUUACUGAAGAAAUCCAGAUGGGUCUGAAUCACC
CUGGUGUUUACCACUGAUCCAAACAUCCGAGUUGGCUACUGCUCCC
AAAUUCCAAACUGUGAUAUGUCACAUGGACAAGAUUGUUAUCGUGG
GAAUGGCAAAAAUUAUAUGGGCAACUUAUCCCAAACAAGAUCUGGAC
UGGCAUGUUCAAUGUGGGACAAGAACAUGGAAGACUUACAUCGUCA
UAUCUUCUGGGAACCAGAUGCAAGUAAGCUGAAUGAGAAUUACUGC
CGAAAUCCAGAUGAUGAUGCUCAUGGACCCUGGUGCUACACGGGAA
AUCCACUCAUUCCUUGGGAUUAUUGCCCUAUUUCUCGUU

b

C-HGF 119 aa
MGNLSQTRSGLACSMWDKNMEDLHRHIFWEPDASKLNENYCRNPDDD
AHGPWCYTGNPLIPWDYCPISRF<u>LNAWPAMGRVIEVSWIIQNQARFVSA</u>
<u>GIIRHHTGTNSCLKDIPTRALMI</u>

Specific aa for C-HGF

c

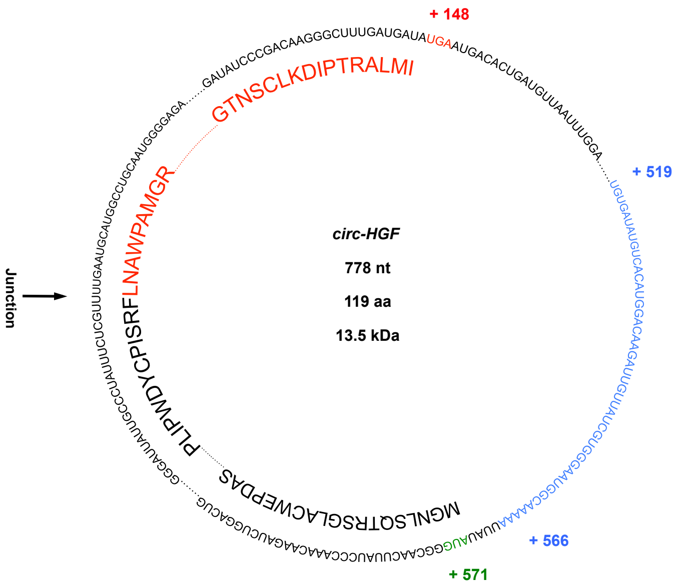

Supplementary Figure S5. a Nucleotide sequence of *circ-HGF* (*hsa_circ_0080914*) RNA. **b** The amino acid sequence of C-HGF. Shown in red are the 49 C-HGF specific aa. **c** *circ-HGF* sequence showing the positions of the back-splicing junction, IRES (blue), start codon (green) and stop codon (red).
