# Supplementary material for: Translation of circHGF RNA encodes an HGF protein variant promoting glioblastoma growth through stimulation of c-MET
Source: J Neurooncol. 2023 May 10;163(1):207–18. doi: 10.1007/s11060-023-04331-5 (PMC10232650; doi:10.1007/s11060-023-04331-5)
Supplement: Supplementary file 7 — Supplementary file7 (PDF 268 KB) [file 11060_2023_4331_MOESM7_ESM.pdf]

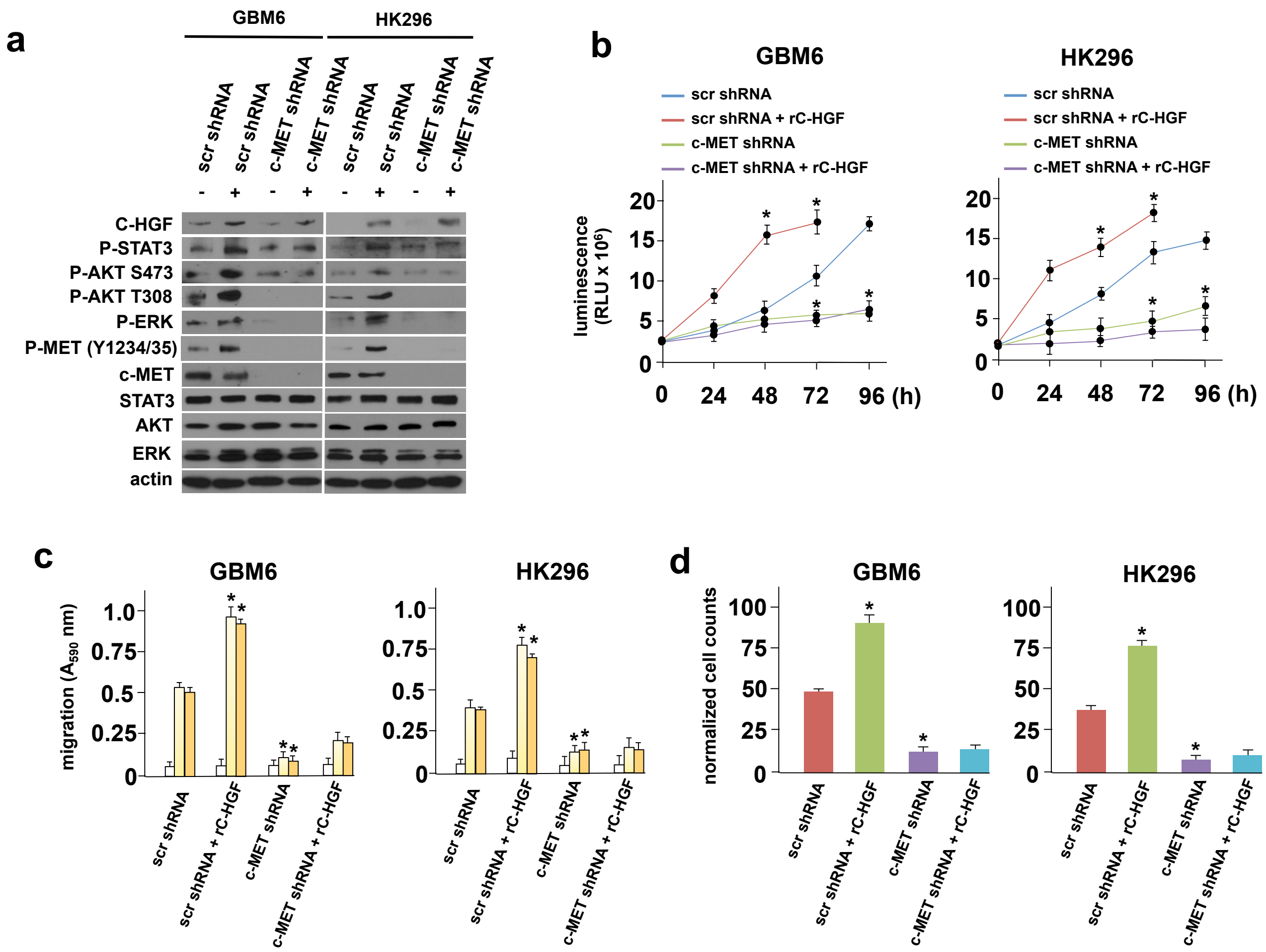

Supplementary Figure S5. Effects of c-MET knockdown on rC-HGF induced cell signaling, proliferation, migration and invasive capacity in GBM6 and HK296 cells. **a** GBM6 or HK296 cells stably expressing either a control nontargeting scramble (scr) sequence or c-MET targeting shRNA were treated (- / +) with rC-HGF (200 ng/ml, 8 h) as shown and immunoblotted for the indicated proteins. **b** Scr shRNA or c-MET shRNA expressing GBM6 or HK296 cells were treated with or without (200 ng/ml) of rC-HGF and proliferation determined via ATP-release assays at the timepoints indicated. Mean ± S.D., *, $p < 0.05$, n = 3. **c** Scr shRNA or c-MET shRNA expressing GBM6 or HK296 cells were placed in Boyden chambers in the absence or presence of rC-HGF (200 ng/ml) and allowed to migrate towards BSA (white bars), vitronectin (light yellow bars) or fibronectin (dark yellow bars). Mean + S.D.., *, $p < 0.05$, n = 3. **d** Invasive potential of scr shRNA or c-MET shRNA expressing GBM6 or HK296 cells in the absence or presence of rC-HGF (200 ng/ml) migrating through Matrigel. Mean + S.D., *, $p < 0.05$, n = 3.
